# Frequency of polymorphisms in the *CYP2C9, VKORC1*, and *CYP4F2* genes related to the metabolism of Warfarin in healthy donors from Cali, Colombia

**DOI:** 10.1097/MD.0000000000034204

**Published:** 2023-07-28

**Authors:** Sebastian Giraldo-Ocampo, Lorena Diaz-Ordoñez, Yisther Katherine Silva-Cuero, Juan David Gutierrez-Medina, Estephania Candelo, Javier A. Diaz, Harry Pachajoa

**Affiliations:** a Centro de Investigaciones en Anomalías Congénitas y Enfermedades Raras (CIACER), Universidad Icesi, Cali, Colombia; b Departamento de Ciencias Básicas Médicas, Facultad de Salud, Universidad Icesi, Cali, Colombia; c Centro de Investigaciones Clínicas, Cali, Colombia; d Genetic Division, Cali, Colombia.

**Keywords:** cytochrome *P*-450 CYP2C9, cytochrome *P*-450 CYP4F2, human, pharmacogenetics, subunit 1 protein, vitamin K epoxide reductase complex, warfarin

## Abstract

Alleles in the *VKORC1, CYP2C9*, and *CYP4F2* genes can influence Warfarin dose requirement. We aimed to determine the frequency of the polymorphisms in these genes in healthy individuals from Cali, Colombia. Observational study where total blood was collected from 107 healthy donors who attended a higher educational institution in Cali, Colombia. Sanger sequencing of exons 2, 3, 5, and 7 of the *CYP2C9* gene; the common promoter region of CYP (rs12777823); exon 11 of *CPY4F2* and the polymorphism c.-1639G > A in the *VKORC1* gene promoter was performed. CYP2C9*2, CYP2C9*3, CYP2C9*8, CYP2C9*9, CYP2C9*11, CYP4F2*3, rs12777823, and VKORC1*2 were detected. The latter had the highest frequency with 80 (74.8%) participants in a heterozygous or homozygous state. The least frequent allele was CYP2C9*11 with only 1 carrier. Combined haplotypes (VKORC1 *1/*2 or *2/*2 and CYP2C9 *1/*2 or *2/*2) were identified in 14 (13.7%) subjects. Both frequencies found in the *VKORC1* and *CYP2C9* alleles were similar to the ones reported for Latin Americans of European and Native American Ancestry. VKORC1*2 allele, the main genetic contributor to Warfarin dosing requirement, was the variant with the highest frequency (74.8% subjects, with a frequency of the alternative allele (A) of 50%). Our findings provide researchers with a greater insight regarding the frequency of common polymorphisms that affect anticoagulation treatment in the Cali (Colombia) population.

## 1. Introduction

Warfarin is a widely prescribed oral anticoagulant used in the treatment and prevention of thromboembolic events, with annual prescriptions typically ranging from 0.5% to 1.5% worldwide.^[1]^ Warfarin is a vitamin k antagonist, which produces its anticoagulant effect through the inhibition of the enzyme vitamin K epoxide reductase.^[2]^ Despite its widespread use, determination of the optimal and safest dosage of Warfarin remains challenging due to its narrow therapeutic window.^[3]^ Even though the introduction of the International Normalized Ratio in the early 1990s allowed for more consistent and universal management of Warfarin therapy,^[2]^ this drug continues to be an important cause of drug-related adverse events, including thromboembolism, ecchymosis, and severe gastrointestinal or intracranial hemorrhage.^[3,4]^ Hence, it is essential to individualize Warfarin dosage due to its potential adverse effects, especially when considering that several factors contribute to the varying response of patients to anticoagulant treatment such as age, ethnicity, body size, vitamin K intake, comorbidities, concomitant drugs, and genetic single nucleotide variants or polymorphisms (SNP).^[5,6]^ The latter accounts for up to the 35% to the observed inter-variability response to Warfarin, and when all the above-mentioned variables are collectively considered along with pharmacogenetic data (genetic variants in drug metabolism-related genes), up to 50% of the variability can be explained.^[7]^

To date, 3 pharmacogenes heavily involved in Warfarin metabolism, have been described^[5]^: cytochrome P450 Family 2 Subfamily C Member 9 (CYP2C9), which is an isoenzyme that degrades S-Warfarin and is 5 to 6 times more biologically active than R-Warfarin;^[8]^ Vitamin K Epoxide Reductase Complex Subunit 1 (VKORC1), which is the target enzyme of Warfarin; and Cytochrome P450 Family 4 Subfamily F Member 2 (CYP4F2), an enzyme catalyzing drug metabolism and associated with variability in Warfarin dose requirements.^[9]^ The information provided by polymorphisms in *CYP2C9* and *VKORC1* genes is clinically very useful and has led to the implementation of pharmacogenetic algorithms, proposed by the International Warfarin Pharmacogenetics Consortium^[10]^ and the US Food and Drug Administration,^[11]^ which demonstrates that pharmacogenetic-guided Warfarin initial dose is superior to clinical algorithms.

Given the importance of genetic variants for anticoagulant treatment in these genes, studies aiming to discover common Warfarin treatment related-SNP frequencies in different populations, including their association with outcomes in patients prescribed with this drug, are crucial. Several studies have been conducted in Europe, Asia, and North America.^[12]^ In the case of Latin America, data are available in Brazil,^[13]^ Peru,^[14,15]^ Puerto Rico,^[15,16]^ Argentina,^[15,17]^ Mexico,^[15,18]^ Cuba,^[15]^ and Colombia.^[19,20]^ Nevertheless, the genetic data reported are usually related to the *CYP2C9* gene, and given the multiethnic conformation of Latin–American countries, more data from different populations are needed to capture possible heterogeneities within countries. In this manner, the pharmacogenomic characterization of the Colombian population remains largely unknown. Thus, the aim of this study was to determine the allelic frequency of common SNP in the *CYP2C9, VKORC1*, and *CYP4F2* genes, associated with Warfarin metabolism in Colombian healthy donors from the city of Cali.

## 2. Methods

### 2.1. Study subjects

This is an observational study where a group of 107 healthy donors was recruited at the Universidad Icesi (Cali, Colombia) between January and March 2019. All subjects were over 18 years of age and recognized themselves as healthy individuals, and therefore, were not on anticoagulant/Warfarin treatment. Convenience sampling was used. This study was approved by the Human Research Ethics Committee of the Universidad Icesi (ID 199), and conducted in accordance with the Declaration of Helsinki. The subjects provided written informed consent before recruitment.

### 2.2. Genotyping of CYP2C9, VKORC1, and CYP4F2 alleles

Anticoagulated total peripheral blood measuring 4 mL was taken in Vacutainer tubes containing EDTA. Total DNA was extracted using the E.Z.N.A.® Tissue DNA Kit (Omega Bio-Tek, Norcross, GA) in accordance with the manufacturer’s instructions. The concentration and purity (260/280 and 260/230 ratios) of the nucleic acids were evaluated with a NanoDrop 2000 spectrophotometer (Thermo Fisher Scientific, Waltham, MA).

Single-stranded Sanger sequencing of *VKORC1* gene promoter, exons 2, 3, 5, and 7 of *CYP2C9,* exon 11 of *CYP4F2*, and the rs12777823 variant in the CYP2C cluster was performed. The DNA was separately amplified by conventional Polymerase Chain Reaction (PCR) to a final volume of 25 μL per reaction, containing, at final concentrations, 1X Green GoTaq Flexi Buffer, 4 mM MgCl_2_, 0.25 µM of each primer, 0.2 mM dNTPs, 1.25 Units of GoTaq® DNA Polymerase (Promega, Madison, WI) and 100 ng of DNA. Primer sequences and their annealing temperatures are detailed in Table 1. The primers used for exons 2 to 3 of *CYP2C9*, rs12777823, and VKORC1*2 were designed using the Primer3 software.^[21]^ The primers for the other regions have already been reported in previous studies.^[22,23]^ The thermocycler protocol was as follows: initial denaturation at 95°C for 5 minutes, denaturation at 94°C for 30 seconds, specific primer annealing temperature for 30 seconds, and extension at 72°C for 45 seconds for 35 cycles, and a final extension for 7 minutes at 72°C.

**Table 1:** Alleles analyzed in the study along with the sequences of the primers used in the PCR to amplify and sequence the regions of the corresponding gene.

Gene	Region	Allele	Primer sequences(5′-3′)	Product size (pb)	Annealing temperature (°C)	Reference
CYP2C9	Exon 2-3	*2, *8, *13, *14, *25	AATCATCATCATGTTTCTATT	746	57	This article
			GAGGACTCATAATGAAAGATA			
	Exon 5	*6, *9, *10	GGAAATGATTATCATCTGGTTAG	642	59	22
			TAGTTTATATTTCTGTGGGCTC			
	Exon 7	*3, *4, *5, *11	GTGCATCTGTAACCATCCTC	428	59	22
			TAAGAGTAGCCAAACCAATCTT			
CYP4F2	Exon 11	*3	AGTCCCGGTCATCTCCCGCCAT	358	58	23
			CGCCAGCCTTGGAGAGACAGACA			
rs12777823	*CYP2C* gene cluster on chromosome 10	rs12777823	GGAGTCTGTGCTGTCTCCAAT	382	62	This article
			GCTGGAAAGTTGAAGATGTCC			
VKORC1	−1639G > A	*2	CACAGACGCCAGAGGAAGAGAG	290	65	This article
			CTCAGCCTCCCAAGTAGTTTGG			

To ensure proper amplification, PCR products were separated by gel electrophoresis with agarose 1% at 100V for 40 minutes, stained with ethidium bromide, and visualized using UV light. Subsequently, after verifying the suitability of PCR products, the amplicons were purified using the E.Z.N.A.® Cycle Pure Kit (Omega Bio-Tek) following the manufacturer recommendations, and sequenced using the BigDye Terminator 3.1 Kit (Thermo Fisher Scientific). The cycle sequencing reaction was performed according to the manufacturer’s instructions. Excess of BigDye terminators was removed using the BigDye XTerminator™ Purification Kit (Thermo Fisher Scientific), and samples were analyzed on the 3500 Genetic Analyzer (Thermo Fisher Scientific). Sequence data were analyzed with the MEGA X software^[24]^ using the GenBank reference sequences of *CYP2C9* (NG_008385), *CYP4F2* (NG_007971), *VKORC1* (NG_011564), and rs12777823 (NC_000010).

A total of 15 SNPs were evaluated to determine the alleles associated to Warfarin treatment: the variant −1639G > A at *VKORC1* gene promoter (VKORC1*2); CYP2C9*2, *8, *13, *14, *25 (exons 2 and 3); CYP2C9*6, *9, *10 (exon 5) and CYP2C9*3, *4, *5, *11 (exon 7) of the *CYP2C9* gene; CYP4F2*3 (exon 11) of *CYP4F2*, and the rs12777823 variant in *CYP2C* gene cluster. Their positions on the respective sequence reference were as follows: c.430C > T (CYP2C9*2), c.1075A > C (CYP2C9*3), c.1076T > C (CYP2C9*4), c.1080C > G(CYP2C9*5), c.818del (CYP2C9*6), c.449G > A (CYP2C9*8), c.752A > G (CYP2C9*9), c.815A > G (CYP2C9*10) c.1003C > T (CYP2C9*11), c.269T > C (CYP2C9*13), c.374G > A (CYP2C9*14), c.353_362del (CYP2C9*14), c.1297G > A (CYP4F2*3), g.94645745G > A (rs12777823), and c.-1639G > A (VKORC1*2).

### 2.3. Warfarin dose requirement

All volunteers were individually analyzed to establish a hypothetical Warfarin dose if they ever need anticoagulant treatment, following the Clinical Pharmacogenetics Implementation Consortium guideline for pharmacogenetics-guided Warfarin dosing: 2017 update.^[25]^ Each participant was assigned a predicted dose: standard dose (StD) 5 to 7 mg/d; low dose (LD) 3 to 4 mg/d; and very low dose (VLD) ≤ 2 mg/d.

### 2.4. Allele frequencies from populations of different ancestry

Data on the frequencies of polymorphisms in individuals from different ancestries were obtained from the Allele Frequency Aggregator project^[26]^ found in the database of Single Nucleotide Polymorphisms (dbSNP, https://www.ncbi.nlm.nih.gov/snp). The sample size of each population was multiplied by 2 to obtain the total number of alleles, and this number was subsequently multiplied by the percentage of the wildtype and alternative alleles to obtain the absolute frequency of each allele in each population.

### 2.5. Statistical analysis

Hardy–Weinberg equilibrium analysis (χ^2^ test with Yates’ correction) of the detected SNP and Fisher exact tests were performed in R programing language using the R Studio software version 4.1.0 and the Hardy–Weinberg Package.^[27]^ Statistical significance was established when the two-tailed test *P* value was lower than .05.

## 3. Results

We included 53 females (49.5%) and 54 males who attended the Universidad Icesi (Cali, Colombia) as students, professors, or employees, with a median age of 24 years (IQR: 22–27.5). Sixty-four participants (60%) were born in the city of Cali and the remaining participants were born in 24 different cities in Colombia but currently living in Cali.

CYP2C9*2, CYP2C9*3, CYP2C9*8, CYP2C9*9, CYP2C9*11, CYP4F2*3, rs12777823, and VKORC1*2 were the alleles found by Sanger sequencing in the 107 subjects included in this study. The 2 most common polymorphisms found were c.-1639G > A (VKORC1*2) in 80 (74.8%) participants (n = 53 [49.5%] and n = 27 [25.2%] in heterozygosis and homozygosis, respectively) and the variant c.1297G > A (CYP4F2*3) in 49 (45.8%) subjects (n = 41 [38.3%] heterozygous and n = 8 [7.8%] homozygous). Among the *CYP2C9* alleles, c.430C > T (CYP2C9*2) was the most frequent variant found in 16 individuals (14%) of whom 15 were carriers and only one in a homozygotic state (Table 2). There were no differences in the SNPs frequencies between males and females (Exact fisher test *P* value > .05). The haplotype frequencies of the 107 subjects included in this study and the allelic frequency found in our study and in individuals from different ancestry, taken from the Allele Frequency Aggregator project, are depicted in Tables 3 and 4, respectively. Regarding VKORC1 and CYP2C9 alleles, 8 subjects (7.5%) were *1/*2 and *1/*2, 1 subject was *1/*2 and *2/*2, and 5 subjects (4.7%) were *2/*2 and *1/*2.

**Table 2:** Genotype of the polymorphism studied in all participants, and divided by gender and city of birth (n = 107). The last column indicates the absolute and relative frequency of the wildtype and alternative allele for each gene (n = 214, 2 alleles per subject), respectively.

Allele	Genotype	All (n = 107)	Male (n = 54)	Female (n = 53)	Cali (n = 64)	Other cities (n = 43)	Total
CYP2C9*2	CC	91 (85)	45 (83.3)	46 (86.8)	54 (84.4)	37 (86)	197 (92.1)
	CT	15 (14)	9 (16.7)	6 (11.3)	10 (15.6)	5 (11.6)	
	TT	1 (0.9)	0	1 (1.9)	0	1 (2.3)	17 (7.9)
CYP2C9*3	AA	98 (91.6)	50 (92.6)	48 (90.6)	59 (92.2)	39 (90.7)	204 (95.3)
	AC	8 (7.5)	3 (5.6)	5 (9.4)	5 (7.8)	3 (7)	
	CC	1 (0.9)	1 (1.9)	0	0	1 (2.3)	10 (4.7)
CYP2C9*8	GG	105 (98.1)	52 (96.3)	53 (100)	63 (98.4)	42 (97.7)	212 (99.1)
	GA	2 (1.9)	2 (3.7)	0	1 (1.6)	1 (2.3)	
	AA	0	0	0	0	0	2 (0.9)
CYP2C9*9	AA	105 (98.1)	54 (100)	51 (96.2)	62 (96.9)	43 (100)	212 (99.1)
	AG	2 (1.9)	0	2 (3.8)	2 (3.1)	0	
	GG	0	0	0	0	0	2 (0.9)
CYP2C9*11	CC	106 (99.1)	53 (98.1)	53 (100)	63 (98.4)	43 (100)	213 (99.5)
	CT	1 (0.9)	1 (1.9)	0	1 (1.6)	0	
	TT	0	0	0	0	0	1 (0.5)
CYP4F2*3	GG	58 (54.2)	32 (59.3)	26 (49.1)	34 (53.1)	24 (55.8)	157 (73.4)
	GA	41 (38.3)	21 (38.9)	20 (37.7)	22 (34.4)	19 (44.2)	
	AA	8 (7.5)	1 (1.9)	7 (13.2)	8 (12.5)	0	57 (26.6)
rs12777823	GG	87 (81.3)	42 (77.8)	45 (84.9)	52 (81.3)	35 (81.4)	194 (90.7)
	GA	19 (17.8)	12 (22.2)	7 (13.2)	12 (18.8)	7 (16.3)	
	AA	1 (0.9)	0	1 (1.9)	0	1 (2.3)	22 (10.3)
VKORC1*2	GG	27 (25.2)	11 (20.4)	16 (30.2)	17 (26.6)	10 (23.3)	107 (50)
	GA	53 (49.5)	26 (48.1)	27 (50.9)	34 (53.1)	19 (44.2)	
	AA	27 (25.2)	17 (31.5)	10 (18.9)	13 (20.3)	14 (32.6)	107 (50)

Alleles in bold are not in Hardy-Weinberg equilibrium.

**Table 3:** Frequencies of haplotypes in the sample analyzed.

VKORC1/CYP2C9	*1/*1	*1/*2	*2/*2	*1/*3	*3/*3	*1/*8	*1/*11	Total
*1/*1	22 (20.6)8	2 (1.9)2	0	3 (2.8)2	0	0	0	27 (25.2)
*1/*2	38 (35.5)19	8 (7.5)2	1 (0.9)	2 (1.9)2	1 (0.9)	2 (1.9)1	1 (0.9)	53 (49.5)
*2/*2	19 (17.8)8	5 (4.7)3	0	3 (2.8)2	0	0	0	27 (25.2)
Total	79 (73.8)	15 (14)	1 (0.9)	8 (7.5)	1 (0.9)	2 (1.9)	1 (0.9)	107

The number in parenthesis represents relative frequency. The number of patients with heterozygous or homozygous CYP4F2*3 is depicted in superscript.

**Table 4:** Allele frequency of the polymorphisms found in the sample, 2 studies from Colombia and in populations of different ancestry. The convention is: absolute frequency wildtype allele/absolute frequency alternative allele (relative frequency of alternative allele). Populations with statistical differences (Fisher exact test *P* value < .05) in the frequency of the respective polymorphism, when compared to the found in this study, are in bold letters.

	VKORC1*2	CYP2C9*2	CYP2C9*3	CYP4F2*3	rs12777823
This study (Cali, Colombia)	107/107 (50)	197/17 (7.9)	204/10 (4.7)	157/57 (26.6)	194/22 (10.3)
Colombia (Bogota)^[19]^	169/121 (41.7)	263/27 (9.3)	276/14 (4.8)	–	–
Colombia (Bogota + other Colombian regions) ^[20]^	112/148 (56.9)	237/21 (8.1)	247/13 (5)	–	–
Worldwide	**256,369/157,798 (38.1**)	220,321/27,230 (11)	564,437/39,238 (6.5)	525,335/210,428 (28.6)	**234,400/45,647 (16.3**)
Latin American 1*	**1210/473 (28.1**)	**1024/151 (12.9**)	1880/120 (6)	2301/722 (23.9)	**523/104 (16.6**)
Latin American 2†	7618/6157 (44.7)	2236/199 (8.2)	11,159/452 (3.9)	**12,963/3508 (21.3**)	4958/689 (12.2)
European	**218,381/140,798 (39.2**)	171,454/23,601 (12.1)	475,966/33,633 (6.6)	439,725/183,114 (29.4)	**192,835/34,564 (15.2**)
African American	**12,157/1502 (11**)	**7638/285 (3.6**)	**18,425/242 (1.3**)	**20,713/2098 (9.2**)	**10,504/3371 (24.3**)
African	**12,655/1532 (10.8**)	**7928/287 (3.5**)	**19,084/251 (1.3**)	**21,505/2126 (9**)	**10,810/3489 (24.4**)
Asian (not south)	**169/1234 (87.9**)	**6425/6 (0.1**)	12,900/607 (4.5)	10,035/3768 (27.3)	**4377/2124 (32.7**)
South Asian	**8026/2057 (20.4**)	**9406/453 (4.6**)	**9090/1181 (11.5**)	**6601/3778 (36.4**)	**168/99 (37.3**)

* Afro-Caribbean Ancestry.

† European and Native American Ancestry.

Given that the city of origin could affect the frequency of SNP, subjects were divided into 2 groups, namely those from Cali (group 1) and those from other cities of Colombia (group 2). Similar frequencies were found between both groups when analyzing carriers or homozygous individuals combined for alleles CYP2C9*2, CYP2C9*3, CYP4F2*3, rs12777823, and VKORC1*2 (Fisher exact test *P* values > .05). Other alleles were not tested given the general low frequency found. Nevertheless, the relative frequency of homozygosis for VKORC1*2 was higher in Group 1 than in Group 2 (32.6% vs 20.3%), while homozygosis for CYP4F2*3 was only found among subjects in Group 1 (Table 2). CYP2C9*8, CYP2C9*9, and CYP2C9*11 alleles were not found in the Hardy–Weinberg equilibrium (n = 8 subjects, Table 2). All subsequent analyses were done with all the individuals together (n = 107).

According to the haplotypes found in this sample, nearly half of the participants (n = 57, 53.3%) would require a standard dose of Warfarin, as opposed to 38% (n = 41) who would require a low or very low dose. Nine participants could not be assigned to a predicted dose, since they presented combined genotypes of CYP4F2*3 and rs12777823 (Table 5).

**Table 5:** Predicted dose of Warfarin requirement by genotype according to CPIC recommendations.

Warfarin requirement		n (%)	Recommended dose (mg/d)
Standard dose		**57 (53.3**)	
	Standard no carriers*	25 (23.4)	5–7
	Carriers CYP4F2*3	24 (22.4)	5.25–7.7
	Carriers rs12777823	8 (7.5)	3.7–6.3
Low dose		**37 (34.6**)	
	Low no carriers*	17 (15.9)	3–4
	Carriers CYP4F2*3	16 (15.0)	3.15–4.4
	Carriers rs12777823	4 (3.7)	2.2–3.6
Very low dose		**4 (3.7**)	
	Very low no carriers*	2 (1.9)	0.5–2
	Carriers CYP4F2*3	2 (1.9)	0.5–2.2
Unknown		**9 (8.4**)	
	Mixed genotype	9 (8.4)	Unknown

Bold values represent the total number of patients requiring each dose.

* No carriers of CYP4F2*3 or rs12777823.

Other additional variants found in the analyzed regions were rs9332129 and rs116236543 in exon 5 of *CYP2C9*; rs9332197 and rs141283168 in exon 7 of *CYP2C9*; rs9332119, rs9332120, rs113044007, rs896332784, and rs9332121 in exons 2 and 3 of *CYP2C9*; and rs3093196 in exon 11 of *CYP4F2*. However, aside from variants rs9332119 (n = 5 subjects, n = 4 from Cali), rs9332120 (n = 10 subjects, n = 3 from Cali), and rs9332121 (n = 3 subjects), all of the other variants were found in only 1 subject each.

## 4. Discussion

There is strong evidence that Warfarin dose is influenced by the VKORC1*2, CYP2C9*2, and CYP2C9*3 alleles, explaining why these alleles have been incorporated in algorithms for the determination of the initial Warfarin dose in clinical settings.^[1]^ The frequencies of the above-mentioned alleles and their impact on Warfarin dosing have been studied in patients and healthy donors of different ancestry, comprising African Americans, Caucasians, Japanese, Han Chinese, Indians, and Hispanics, among others.^[3,15,23,28]^ The results of these previous studies highlight the differences in frequency of these alleles, and therefore, in the general requirement of Warfarin dose among individuals from different geographical regions. In this manner, it is necessary to determine the prevalence of these alleles and haplotypes in individuals of different ancestry, but even within genetically heterogeneous populations, such as in Latin America. The present study revealed that the c.-1639G > A in the *VKORC1* gene promoter was the most common variant in a population sample from Cali (Colombia) comprised of 107 subjects, with 80 (74.8%) participants in a heterozygous or homozygous state, and the frequency of the alternative allele (A) was 50%. In contrast, c.1003C > T (CYP2C9*11) was the least frequent variant with only one heterozygous individual, while CYP2C9*2 and CYP2C9*3 represented 14.9% and 8.4%, respectively. These results are consistent with the frequencies found in the *VKORC1* promoter, CYP2C9*2, and CYP2C9*3 in patients undergoing Warfarin treatment in Bogota, the capital of Colombia.^[19,20]^ Our findings indicate that despite the slight difference found between these 2 cities, the frequencies of the analyzed alleles were similar.

The genetic frequencies of VKORC1*2, CYP2C9*2, CYP2C9*3, CYP4F2*3, and rs12777823 found in our samples are similar to the ones shown for Latin Americans of European and Native American Ancestry and European populations; however, there are distinct differences with Latin Americans of Afro–Caribbean ancestry or African, African American, and South Asian populations (Table 4). In the particular case of VKORC1*2 frequency, a comparison with other populations (Table 4) demonstrated statistical differences with all but Latin Americans of European and Native American Ancestry, indicating that this allele presents an important prevalence variability even within the Latin–American populations. These results are important because *VKORC1* is the main genetic factor that influences the inter-individual response to Warfarin dosing, with VKORC1*2 explaining up to 47% inter-individual response in Caucasians and up to 30.3% in Hispanics.^[29]^ In contrast, *CYP2C9* is the second strongest genetic factor associated with Warfarin dose, accounting for approximately 2% to 27% of variability (up to 8% in Hispanics) when CYP2C9*2 and CYP2C9*3 are combined.^[29]^ The frequency of these 2 alleles was similar when compared with European and the Latin–American populations except for CYP2C9*2 in Latin Americans of Afro-Caribbean ancestry. Frequencies of these 2 alleles, and CYP4F2*3 were similar to the worldwide frequency (Table 4).

Association between CYP4F2 genotype and the required Warfarin dose requires further investigation. Although the variability explained by the alleles of this gene ranges between 1% and 11%,^[30,31]^ these genetic changes have yet to be considered in clinical practice, particularly given the dominant role of *VKORC1* and *CYP2C9* genes. Furthermore, CYP4F2*3 seems to be a modifier of the Warfarin dose requirement when certain haplotypes of these 2 genes are present, especially in some populations such as Asians.^[30]^ The rs12777823 follows a similar scenario as the one previously described. While individuals of European or Asian ancestry with CYP4F2*3 alleles require a modest increase in Warfarin dose, individuals of African ancestry, and at least one copy of the rs12777823 variant require lower dosing of the drug.^[32]^ Nevertheless, given the lack of studies on the genotypes of these genes in treating Colombian populations with Warfarin, no conclusion can be drawn. The frequency of rs12777823 found in this study is different from the one reported in other populations, including Latin Americans of Afro-Caribbean ancestry except Latin Americans of European and Native American Ancestry (Table 4).

According to the frequencies of haplotypes found, most participants in the present study (53.3%) would require a standard dose of Warfarin (5–7 mg/d) given their wild type or heterozygous genotypes in the CYP2C9*2, CYP2C9*3, and VKORC1*2 alleles. However, the calculated requirement would be higher or lower in the presence of CYP4F2*3 or rs12777823 alleles, respectively (Table 5). Furthermore, 38% of healthy volunteers would require a low or a very low Warfarin dose (0.5–4.4 mg/d). Therefore, special care is essential in cases where these individuals require anticoagulant treatment given that a standard initial dose of this drug could cause bleeding. In contrast, 6 participants (3.74%) were heterozygous to both CYP4F2*3 and rs12777823, implying a dose reduction between 10% to 25% and a dose increase of 5% to 10%, respectively. Due to the absence of guidelines to include both alleles in the same patient, the predicted initial dose was not calculated in these subjects nor for 3 subjects with CYP2C9*8 and CYP2C9*11 associated with CYP4F2*3 and rs12777823. Therefore, although these alleles are not the main genetic factor influencing Warfarin metabolism, more studies evaluating their influence on the initial dose are required, including the potentially predominant effect that these alleles can exert on another alleles.

In the future, the use of a panel-based preventive approach before prescribing medications with narrow therapeutic windows and potential high-risk side effects will require genotype information on hundreds of pharmacogenes that should be readily available in the patient’s medical history to guide the subsequent drug therapy.^[31]^ In our population, it is necessary to determine the frequency of the polymorphisms in pharmacogenes, previously reported in literature, prior to implementing these approaches due to the high-grade genetic variability. Our results support this approach due to the differences found with other populations (Table 4). When compared to the findings in other populations, the differences identified in the frequency of the polymorphisms in the present study may reflect the heterogeneous genetic background given by the 3 Latin–American parental populations (European, African, and Native Amerindians) with a wide degree and diverse patterns of genetic admixture. The knowledge of these data would enable the selection of the appropriate therapeutic approach and dosing strategy for different drugs (in this particular case, Warfarin). In addition, knowing the frequency of different polymorphisms in pharmacogenes will allow the long-term determination of which types of drugs may pose a risk to some patients and also the types and correct dosages of drugs. However, to reach that point, it is critical to gain a greater insight regarding the prevalence of different polymorphisms in pharmacogenes in healthy donors from different populations.

We acknowledge that there are several limitations to this study. For instance, the present study did not include a group undergoing anticoagulant treatment, and thus could not evaluate whether the polymorphisms found could affect treatment and to what degree. Furthermore, the diverse genetic background of the sample is another limitation, while all participants were recruited in the same place. In addition, the present study employed Sanger sequencing which is an approach that is not commonly used to determine the alleles analyzed (although other genetic variants can be found through this methodology, which could be affecting the treatment response). Finally, this study did not determine the ancestry of the subjects included.

In conclusion, our results facilitate a greater understanding of the genotype spectrum in enzymes related to Warfarin metabolism in Colombia, contributing to future strategies that enable the improved stratification of patients for more cost-effective treatments by reducing side effects related to underprescription (thromboembolic events) or overprescription (hemorrhagic events) of Warfarin, which a leading cause of hospitalization. This study also demonstrated that the frequency of VKORC1*2, CYP2C9*2, and CYP2C9*3, which are the most important genetic contributors to Warfarin dose requirement, in a sample from Cali (Colombia), are different from other populations of different ancestries, rendering necessary the evaluation of different Colombian populations for determining the real frequency of these genetic changes.

## Acknowledgments

The authors would like to thank Equipos y Laboratorios de Colombia for their kind donation of a critical reactive for Sanger sequencing of the samples, and for promoting science and research and helping us to build a better future.

## Author contributions

**Conceptualization:** Lorena Diaz-Ordoñez, Estephania Candelo, Harry Pachajoa.

**Data curation:** Sebastian Giraldo-Ocampo, Lorena Diaz-Ordoñez, Yisther Katherine Silva-Cuero, Juan David Gutierrez-Medina.

**Formal analysis:** Sebastian Giraldo-Ocampo, Lorena Diaz-Ordoñez.

**Funding acquisition:** Lorena Diaz-Ordoñez, Harry Pachajoa.

**Investigation:** Lorena Diaz-Ordoñez, Yisther Katherine Silva-Cuero, Juan David Gutierrez-Medina, Javier A. Diaz, Harry Pachajoa.

**Methodology:** Lorena Diaz-Ordoñez, Yisther Katherine Silva-Cuero, Juan David Gutierrez-Medina, Javier A. Diaz.

**Project administration:** Yisther Katherine Silva-Cuero, Harry Pachajoa.

**Supervision:** Sebastian Giraldo-Ocampo, Lorena Diaz-Ordoñez, Yisther Katherine Silva-Cuero, Harry Pachajoa.

**Writing – original draft:** Sebastian Giraldo-Ocampo, Javier A. Diaz.

**Writing – review & editing:** Sebastian Giraldo-Ocampo, Lorena Diaz-Ordoñez, Yisther Katherine Silva-Cuero, Juan David Gutierrez-Medina, Estephania Candelo, Javier A. Diaz, Harry Pachajoa.
